# Evaluating tropical phytoplankton phenology metrics using contemporary tools

**DOI:** 10.1038/s41598-018-37370-4

**Published:** 2019-01-24

**Authors:** John A. Gittings, Dionysios E. Raitsos, Malika Kheireddine, Marie-Fanny Racault, Hervé Claustre, Ibrahim Hoteit

**Affiliations:** 1 Department of Earth Science and Engineering, King Abdullah University of Science and Technology (KAUST), Thuwal, 23955-6900 Saudi Arabia; 2 Remote Sensing Group, Plymouth Marine Laboratory (PML), The Hoe, Plymouth, PL1 3DH United Kingdom; 3 National Centre for Earth Observation (NCEO), Plymouth Marine Laboratory (PML), The Hoe, Plymouth, PL1 3DH United Kingdom; 4 Department of Biology, National and Kapodistrian University of Athens, Athens, Greece; 5 Red Sea Research Centre, Biological and Environmental Science and Engineering Division, King Abdullah University of Science and Technology (KAUST), Thuwal, 23955-6900 Saudi Arabia; 6 Marine Optics and Remote Sensing Laboratory, Laboratoire d’Océanographie de Villefranche, Villefranche-sur-Mer, France

## Abstract

The timing of phytoplankton growth (phenology) in tropical oceans is a crucial factor influencing the survival rates of higher trophic levels, food web structure and the functioning of coral reef ecosystems. Phytoplankton phenology is thus categorised as an ‘ecosystem indicator’, which can be utilised to assess ecosystem health in response to environmental and climatic perturbations. Ocean-colour remote sensing is currently the only technique providing global, long-term, synoptic estimates of phenology. However, due to limited available *in situ* datasets, studies dedicated to the validation of satellite-derived phenology metrics are sparse. The recent development of autonomous oceanographic observation platforms provides an opportunity to bridge this gap. Here, we use satellite-derived surface chlorophyll-a (Chl-a) observations, in conjunction with a Biogeochemical-Argo dataset, to assess the capability of remote sensing to estimate phytoplankton phenology metrics in the northern Red Sea – a typical tropical marine ecosystem. We find that phenology metrics derived from both contemporary platforms match with a high degree of precision (within the same 5-day period). The remotely-sensed surface signatures reflect the overall water column dynamics and successfully capture Chl-a variability related to convective mixing. Our findings offer important insights into the capability of remote sensing for monitoring food availability in tropical marine ecosystems, and support the use of satellite-derived phenology as an ecosystem indicator for marine management strategies in regions with limited data availability.

## Introduction

In tropical oceans, phytoplankton constitute a direct food source for coral reef fauna and pelagic larvae^1–4^, whose survival ultimately contributes to healthy, diverse marine ecosystems. This translates to economic support, services and well-being for maritime nations via fisheries and tourism^5^. Phenology characterises the timing of phytoplankton growth periods and is an integral component controlling the structure of marine food webs and marine ecosystem functioning^6,7^. Alterations to phytoplankton phenology may influence the survival of higher trophic levels due to variations in the timing of food availability^8–10^. Thus, monitoring phenology at seasonal and interannual timescales is necessary for the establishment of management strategies in tropical oceans and associated coral reef ecosystems. Phenology metrics, including the timing of phytoplankton growth initiation, maximum amplitude, termination and duration, are referred to as ‘ecological indicators’, representing objective and quantitative measurements that can be utilised to evaluate the condition of marine ecosystems and their response to environmental change^11–14^.

Ocean-colour remote sensing is currently the only method providing continuous, long-term (~20 years), synoptic time series of phytoplankton abundance (indexed by chlorophyll-a [Chl-a] concentration), from which phytoplankton phenology metrics can be computed^15^. However, remotely-sensed Chl-a observations are representative of the surface oceanic layer (~first optical depth), rather than being indicative of the complete vertical phytoplankton distribution within the water column. In particular, stratified tropical ecosystems are characterised by the presence of Subsurface Chl-a Maxima (SCM) that cannot be detected by satellites. To date, attempts to validate satellite-based estimates of phytoplankton phenology with *in situ* measurements remain sparse, primarily due to the lack of continuous, spatially extensive observations^13,16^. The aforesaid limitations of satellite-derived datasets may discourage researchers from utilising remotely-sensed information in ecosystem management schemes. Oceanographic multi-platforms could bridge this gap and provide the necessary information needed to assess the potential of satellite remote sensing in retrieving phenology indices, and also, enable a more holistic quantification of phenology over the whole water column.

Adopting an innovative approach, we synergistically utilise satellite-derived Chl-a observations with data from an autonomous Biogeochemical-Argo float (BGC-Argo float) to evaluate (1) the capability of remote-sensing data to estimate phytoplankton phenology metrics in a typical tropical marine ecosystem – the northern Red Sea; and (2) extend the phenological analysis to the part of the upper water column that is not seen by satellites. We corroborate surface signatures detected by satellites by investigating the physical mechanisms that control vertical phytoplankton dynamics.

## Results

### Comparing phenology metrics from satellite and BGC-Argo datasets

**Figure 1:**
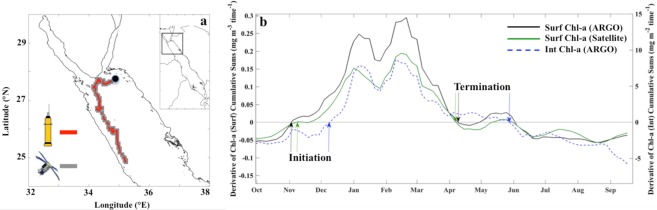
(**a**) Map displaying the track of the PROVOR BGC-Argo float (red circles) and corresponding satellite (OC-CCI) matchups (grey-shaded squares) in the northern Red Sea. A total of 139 vertical profiles were analysed between September 30^th^ 2015 and September 27^th^ 2016). (**b**) Time series displaying the derivative of the cumulative sums of Chl-a anomalies used to identify the timing of phenology metrics (initiation and termination) for the satellite and BGC-Argo datasets. The horizontal grey line located at zero highlights the transition between increasing/decreasing trends in the cumulative sums of Chl-a anomalies (e.g. when Chl-a concentrations rise above/below the phenology threshold criterion, see Materials and Methods).

To evaluate the capability of satellite-derived Chl-a observations for the computation of phytoplankton phenology, we directly compare phenology metrics computed using satellite (OC-CCI) and BGC-Argo Chl-a datasets in the Red Sea (Fig. 1a). We refer to the time series of surface Chl-a concentrations from satellites and the BGC-Argo float as Chl[_Sat-Surf_] and Chl[_Argo-Surf_] respectively, whilst Chl[_Argo-Int_] refers to the time series of integrated BGC-Argo Chl-a over the euphotic depth (see Materials and Methods). First, it is worth noting that Chl[_Sat-Surf_] exhibits a significant correlation with Chl[_Argo-Surf_] and Chl[_Argo-Int_] (n = 154, ρ = 0.90, p < 0.00001 and n = 154, ρ = 0.71, p < 0.00001 respectively), highlighting the strong coherence between the BGC-Argo and satellite datasets. Phenology metrics derived from the two datasets match remarkably well (Fig. 1b).

The initiation of the main phytoplankton growth period as seen from Chl[_Argo-Surf_] and Chl[_Sat-Surf_] occurs in autumn, during late October and early November respectively, with a difference of one five-day period between the two phenology estimates. The initiation of Chl[_Argo-Int_] occurs approximately one month later near the beginning of December. During the main phytoplankton growth period, two prominent peaks are apparent in early January and late February across the three Chl-a datasets. The timings of termination for Chl[_Argo-Surf_] and Chl[_Sat-Surf_] are almost identical and occur in early April, whilst the termination of Chl[_Argo-Int_] occurs ~1.5 months later. In accordance with these timings, Chl[_Argo-Surf_] and Chl[_Sat-Surf_] are characterised by main phytoplankton growth periods with approximately the same duration (~5 months), in contrast to Chl[_Argo-Int_] which is ~2–3 weeks longer.

### Seasonal succession of satellite-derived Chl-a and BGC-Argo vertical profiles

**Figure 2:**
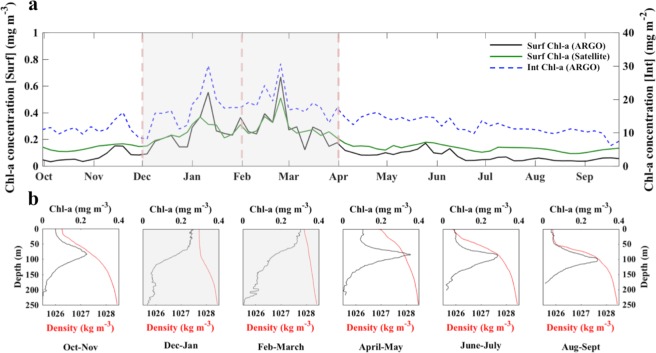
(**a**) Seasonal time series of surface and integrated Chl-a concentrations. The black and green lines represent surface BGC-Argo Chl-a concentrations (averaged over the first optical depth) and satellite-derived surface Chl-a concentrations respectively. The blue-dashed line corresponds to integrated Chl-a concentrations (integrated over the mean euphotic depth of the time series). (**b**) Average bi-monthly vertical profiles of BGC-Argo Chl-a concentrations (black line) and density (red line). The grey panels highlight the main phytoplankton growth period (December–March). We note that the number of profiles used to compute each bi-monthly average varied due to the fluctuating sampling frequency of the BGC-Argo float during its deployment.

To elucidate how satellite-derived surface Chl-a seasonality relates to the vertical dynamics of the water column, we present seasonal time series of the three Chl-a datasets, alongside bi-monthly averages of BGC-Argo Chl-a concentration and density profiles (Fig. 2).

Overall, Chl[_Sat-Surf_] exhibits similar patterns of variability to the BGC-Argo time series (Chl_[Argo-Surf]_ and Chl_[Argo-Int]_). Three distinct phases of the Chl-a seasonal succession are observed. First, a period of low, but increasing, Chl-a concentrations occurs in autumn (October–November), coinciding with the observed timing of phytoplankton growth initiation for the Chl[_Argo-Surf_] and Chl[_Sat-Surf_] time series (Fig. 1b). The initiation of Chl[_Argo-Int_] is detected in early December (Fig. 1b), although Chl-a concentrations also exhibit a transient increase during November (Fig. 2a). Following this, the main phytoplankton growth period is apparent during winter (December –April, grey-shaded panels) and is characterised by an overall increase in Chl-a and two distinct peaks occurring at the beginning of January and in late February. Finally, paralleling the onset of termination for surface Chl-a in early April, and integrated Chl-a in late May, a period of reduced Chl-a concentrations begins in spring and continues throughout summer (June–September, Figs 1b and 2a).

Corresponding *in situ* BGC-Argo vertical profiles reveal a distinct vertical seasonal succession that reflects the seasonal Chl-a time series (Fig. 2b). In autumn (October–November), Chl-a profiles reveal the presence of a SCM located at ~75 metres, whilst density profiles indicate the position of a moderate pycnocline. During the main winter growth period (period of high surface Chl-a, grey-shaded panels, Fig. 2a), vertical profiles highlight the complete erosion of the SCM. Chl-a concentrations are substantially higher and homogenous in the upper mixed layer, before decreasing with depth (Fig. 2b). Density profiles also reveal the increasing homogeneity of the upper water column, particularly during February/March, when the density gradient is very weak in the 0–250 m layer. Vertical profiles during spring (April–May) highlight the re-establishment of the SCM, which has the highest magnitude detected throughout the year (~0.35 mg m^−3^), and the presence of a small pycnocline, coinciding with an overall decrease in surface density (Fig. 2b). Summer vertical profiles portray a further decrease in density and the pycnocline begins to exhibit a stronger stratification gradient (Fig. 2b), associated with a progressive deepening of the SCM (~100 m in August–September).

### Links between satellite phenology metrics and physical mechanisms

**Figure 3:**
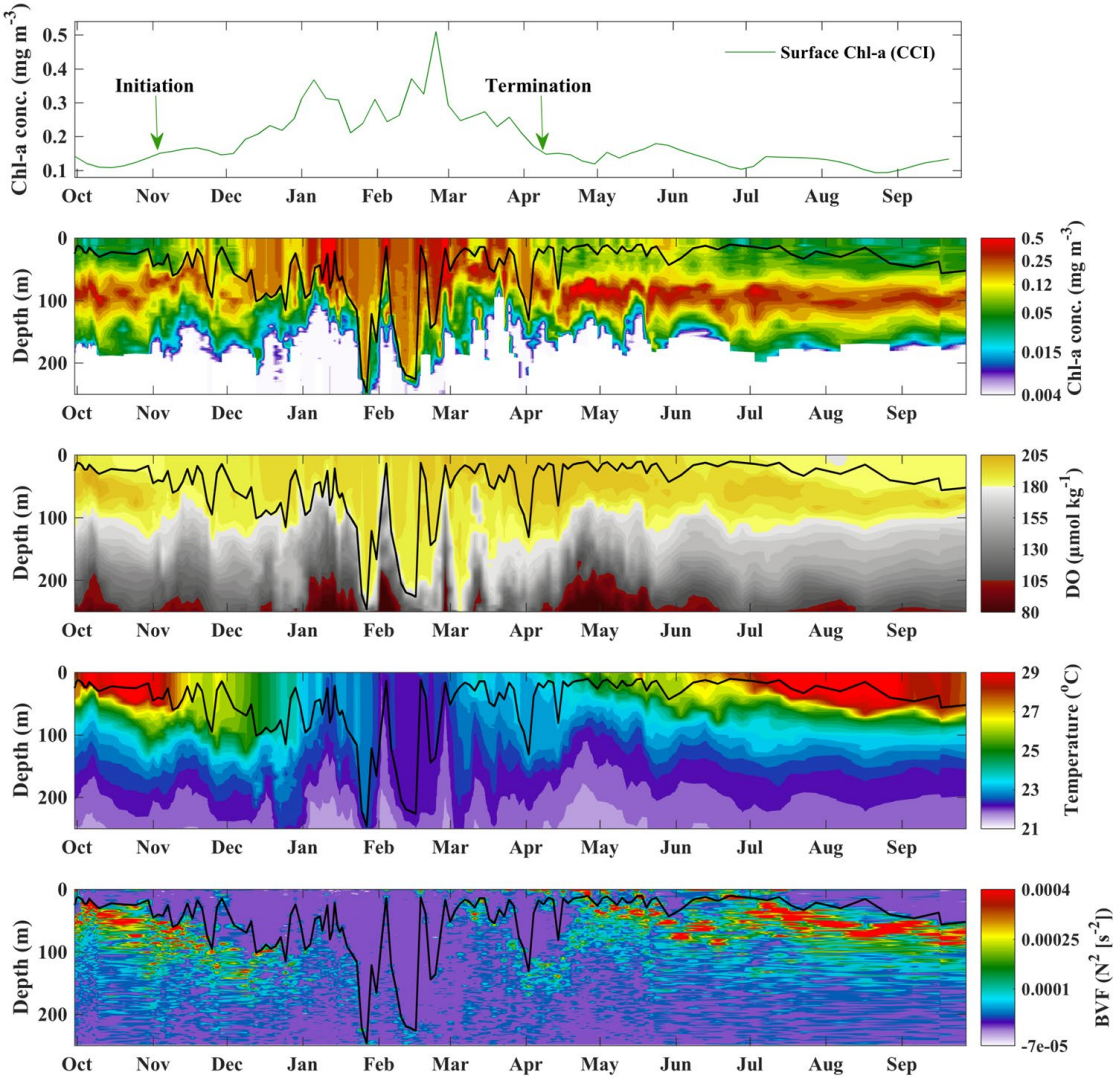
Time series of satellite-derived surface Chl-a concentrations and vertical profiles of BGC-Argo Chl-a concentration, Dissolved Oxygen (DO), temperature and the Brunt–Väisälä Frequency (BVF, an index of stratification), for the period spanning September 30^th^ 2015–September 27^th^ 2016. The green arrows in the first panel display the timings of bloom initiation and termination based on satellite-derived surface Chl-a concentrations. The black line in each panel represents the Mixed Layer Depth (MLD).

We have shown that phenology metrics computed using observations from the two platforms are markedly similar. The seasonal cycle of satellite-derived Chl-a exhibits a strong coherence with *in situ* vertical Chl-a profiles. We further investigate links between satellite-derived phenology and physical processes by analysing the vertical seasonal succession of Chl-a, Dissolved Oxygen (DO), temperature, and the Brunt–Väisälä Frequency (BVF, an index of stratification, Fig. 3).

The timing of Chl[_Sat-Surf_] initiation in early November (Fig. 3), is concurrent with an abrupt deepening of the MLD from ~25 to 50 m, higher surface Chl-a concentrations (~upper 50 m) and a reduction in the BVF. A gradual increase in Chl[_Sat-Surf_] co-occurs with a steady deepening of the MLD until mid-December, when the MLD has reached ~100 metres, coinciding with elevated Chl-a in the mixed layer and the erosion of the SCM (Fig. 3). Accompanying this, in mid-December, the mixed layer exhibits increased levels of DO, a progressive reduction in temperature, and an overall decrease in stratification. The peaks observed in the Chl[_Sat-Surf_] time series during January and February are matched by a striking increase in Chl-a (>0.5 mg m^−3^), the deepening of the MLD to depths of 100–250 metres, and the presence of oxygenated waters (DO, Fig. 3) throughout the mixed layer. Temperature is homogeneous within the mixed layer during these strong mixing events and the BVF accentuates the relative weakening/strengthening of stratification. The termination of the satellite-derived main phytoplankton growth period in early April coincides with a shallower MLD (~25 metres), reduced surface Chl-a concentrations and the re-establishment of the SCM (Fig. 3). DO remains relatively high at the time of termination and temperatures in the mixed layer exhibit an overall increase, continuing to warm throughout spring and summer. The termination of the phytoplankton growth period also overlaps with high values of the BVF (increased stratification) in the shallow mixed layer.

## Discussion

Ocean-colour remote sensing is currently the only platform from which synoptic estimates of phytoplankton phenology – an important ecosystem indicator – can be acquired. Yet, prior to this analysis, research dedicated to the substantiation of satellite-derived phenology metrics was limited. Our study demonstrates that satellite-derived phenology in a typical tropical ecosystem (the northern Red Sea) is consistent with estimates attained using an *in situ* BGC-Argo float dataset, and also appears to be representative of vertical water column dynamics.

Initiation of the main phytoplankton growth period, based on surface Chl-a concentrations from both satellite and BGC-Argo datasets, occurs near-synchronously in late October/early November (Fig. 1b), coinciding with a smaller SCM (in comparison to spring/summer) and an apparent increase in Chl-a concentrations within the surface layer (Figs 2b and 3). The northern Red Sea experiences colder atmospheric conditions and stronger air-sea heat fluxes at the onset of winter, which generates convection events and vertical mixing within the water column^17–24^. In this respect, biological dynamics in the northern Red Sea follow a regime that is analogous to what is typically observed in other tropical marine ecosystems, where the re-distribution of nutrients from the deeper layers is the dominant factor controlling phytoplankton growth^25^. The enhancement of Chl-a captured by satellite sensors in early November (Figs 1b and 3) may represent the initial erosion of the SCM and the redistribution of Chl-a and nutrients to the surface layer. Supporting this, elevated *in situ* surface Chl-a concentrations and the presence of a diminished SCM are paralleled by increased density at the surface (Fig. 2b), and an overall reduction in upper layer stratification, in comparison to summer (Figs 2b and 3). This is analogous with the results of *Lavigne et al*.^26^, who revealed that vertical Chl-a profiles across the Mediterranean (Ionian Sea) exhibit a ‘modified SCM’ shape in early winter, when Chl-a concentrations are higher in the surface layer and peak just below the base of the mixed layer (as can be observed in late October in Fig. 3). The aforementioned authors attributed this type of profile shape to vertical mixing in the upper layer that erodes the SCM. Our results are also consistent with those of *Calbet et al*.^27^, who revealed that the main seasonal phytoplankton growth period in the central Red Sea is likely to be initiated when nutrients are entrained into the upper water column following a deepening of the mixed layer.

The initiation of Chl[_Argo-Int_] with the adopted metrics begins in early December. As Chl[_Argo-Int_] represents Chl-a values integrated over the euphotic depth, we suggest that the one month lag observed in the initiation of Chl[_Argo-Int_] could relate to the amount of time required for vertical mixing to reach depths where there are abundant deposits of subsurface nutrients that can stimulate growth within the entire euphotic zone. Supporting this, we note that the timing of Chl[_Argo-Int_] initiation in early December appears to coincide closely with a deepening of the MLD to ~100 metres and an increase in Chl-a concentrations throughout the upper 100 metre later (Figs 1b and 3), emphasising the potential occurrence of a large mixing event that may have re-distributed nutrients throughout the euphotic layer.

The Chl[_Sat-Surf_] increase observed during the main phytoplankton growth period (December – April) is reflected by an enhancement in both surface and integrated BGC-Argo Chl-a (Figs 2 and 3). During this period, there is a clear intensification of vertical mixing and phytoplankton growth, particularly during January and February, as indicated by weaker density gradients, an overall deepening of the mixed layer (up to ~250 metres), the presence of colder, oxygenated waters and a significant reduction in vertical stratification (Figs 2b and 3). Consistent with our analysis, maximum Chl-a concentrations in the northern Red Sea have previously been reported to occur between January and March as a result of convection-related vertical mixing^17,18,23,24,28^. The timing of prominent peaks in January and February, as evidenced by higher Chl-a concentrations (~0.5 mg m^−3^) within the mixed layer, is well-represented by the Chl[_Sat-Surf_] time series (Fig. 3). These Chl-a peaks occur during periods when the BGC-Argo float passes through areas characterized by deep mixed layers and colder temperatures (Supplementary Fig. 1). In such areas, we suggest that strong convection-driven vertical mixing^24^, and the subsequent redistribution of nutrients from deeper layers, sustains increased levels of phytoplankton growth (Fig. 3). A subsequent shallowing of the MLD, observed immediately following these Chl-a peaks, may be explained by the fact that the BGC-Argo float is transported out of the convection area. Alternatively, cyclonic eddies have been reported to form frequently at ~27°N in the western region of the northern Red Sea^19,21,29–31^ and have been previously associated with the rapid shoaling of the mixed layer^32^.

The timing of Chl[_Sat-Surf_] termination in early April coincides with that of Chl[_Argo-Surf_], paralleling a substantial reduction of *in situ* Chl-a concentrations within the surface layer and the re-establishment of the SCM (Figs 1b and 2). The coincident occurrence of warmer temperatures, a shallower MLD and increased stratification indicates that overall, satellite-derived Chl-a appears to accurately capture the cessation of vertical mixing and the resultant diminished supply of nutrients into the upper euphotic layer. Thus, we hypothesise that once vertical mixing ceases, nutrients within the upper euphotic layer will be rapidly consumed, surface phytoplankton abundance will decrease to levels observed prior to the main growth period, and the SCM will re-form as phytoplankton begin to grow where there is an optimal combination of two diverging resource gradients: light from the surface and nutrients diffused from below^33^ (Figs 2 and 3).

It is evident that Chl[_Argo-Int_] concentrations display a marked decrease (alongside Chl[_Sat-Surf_] and Chl[_Argo-Surf_]) in mid February (Fig. 2a), before plateauing and remaining slightly above the threshold criterion throughout April and May (Fig. 1b). However, the termination of Chl[_Argo-Int_] seems to be delayed by ~2 months. Interestingly, Chl-a concentrations at the SCM during April and May are the highest observed throughout the time series (Figs 2b and 3). We hypothesise that the delayed termination of integrated Chl-a concentrations could be explained by the seasonal dynamics of the SCM in relation to light availability. Following the peak of the main phytoplankton growth period between February and March, we propose that light attenuation in the upper euphotic layer decreases in parallel with Chl-a concentrations. Subsequently, the SCM deepens in response to increased light availability (clearer waters) and becomes closer to the nutricline, leading to an enhancement in phytoplankton biomass. Note that a brief discussion on the potential effects of photoacclimation can be found in the Potential biases section (see Materials and Methods). From June onwards, the magnitude of the SCM decreases, presumably as nutrients at the nutricline are gradually consumed, and thus, less biomass is sustained. We also note that the potential impact of grazing should not be ignored and previous studies in the northern Red Sea have documented the role of zooplankton grazing on phytoplankton biomass^34^. However, as phytoplankton dynamics in the northern Red Sea are suggested to be strongly bottom-up controlled^35^, we speculate that the termination of the main phytoplankton growth period observed in early April is primarily representative of nutrient limitation.

Using a biogeochemical dataset, acquired by an autonomous BGC-Argo float deployed in a relatively unexplored tropical ecosystem – the Red Sea – we demonstrate that remotely sensed surface phenology matches very closely with phenology metrics derived from an *in situ* Chl-a dataset (within the same 5-day period). Satellite-derived surface phenology successfully captures the commencement and culmination of convection-driven vertical mixing, which is the predominant mechanism affecting nutrient availability in the northern Red Sea. Although previous studies have demonstrated the importance of float-based measurements for the validation of satellite ocean-colour products in subtropical waters^36^, to our knowledge, this study comprises the first float-based assessment highlighting the capability of satellite sensors for retrieving phytoplankton phenology in a tropical marine ecosystem.

The timing of food availability in tropical marine ecosystems may be altered under future scenarios of climate warming, potentially having far-reaching impacts on higher trophic levels, reef-dwelling organisms, and coastal fisheries that are an invaluable economic resource in tropical regions. With the consideration that there are presently two decades of satellite data available, the ability to now retrieve representative estimates of surface phenology synoptically from space, at interannual timescales, is paramount for monitoring how tropical ecosystems and their associated coral reef habitats respond to global climate change. Additionally, in data-limited regions where *in situ* sampling efforts are sparse or not possible, satellite-derived phytoplankton phenology is likely to become a fundamental factor influencing the effective design and implementation of future ecosystem management strategies.

## Materials and Methods

### BGC-Argo float data

A NKE CTS4 PROVOR float (World Meteorological Organization, #6901573 http://argo.jcommops.org) equipped with biogeochemical and bio-optical sensors was deployed in the northern Red Sea in September 2015 at 33.73°N and 27.66°E. The float acquired vertical profiles of biogeochemical and optical parameters during its vertical ascent from a maximum parking depth of 1000 m. It was programmed to surface at noon, over time periods varying from 1, 2, 5 or 10 days, depending on the mission’s specifications. Based on the float’s sampling track, a total of 139 vertical profiles were analysed between September 30^th^ 2015 and September 27^th^ 2016, spanning a latitudinal range of approximately 4° (Fig. 1a).

Measurements of pressure, temperature and salinity were obtained via a Seabird standard conductivity-temperature-depth profiler (CTD, model SBE 41CP). Dissolved Oxygen (DO) concentrations were determined using an Aanderaa Optode sensor (model 4330). Vertical profiles of Chl-a fluorescence were acquired using a WET Labs ECO Puck Triplet sensor, whilst radiometric measurements, including Photosynthetically Available Radiation (PAR), were acquired by an OC4 Satlantic radiometer. Following standard procedures for Argo data management^37^, each profile was then quality-controlled using methods that have been specifically developed for each parameter (^38–41^ and references therein). Briefly, vertical profiles of Chl-a were adjusted for non-zero deep values, and corrected for non-photochemical quenching using an empirical method for shallow-mixing waters according to *Xing et al*.^42^. The uncertainties regarding this method (XB18) are provided in their Table 2. Following *Roesler et al*.^43^, the community-established calibration bias of 2 for the WET Labs ECO fluorescence sensor was applied to *in situ* fluorometric Chl-a measurements. After processing, we corroborated Chl-a data by comparing the first vertical profile acquired during the float’s deployment, with a nearby profile of Chl-a concentration obtained by a CTD cast taken on the day before the BGC-Argo deployment (R/V Thuwal, September 29^th^ 2015). The CTD Chl-a measurements were obtained using a similar type of WET Labs ECO Puck Triplet sensor, which was calibrated using High Performance Liquid Chromatography (HPLC) measurements acquired from multiple cruises conducted across the Red Sea^44^. Visual comparison of the two profiles revealed that they were highly similar with regards to their range of Chl-a concentrations, shape and magnitude, providing us with confidence that Chl-a concentrations measured by the BGC-Argo float are representative of the region (figure not shown). DO measurements were corrected by applying a factor of 1.06 to each profile based on the comparison between the surface percent oxygen saturation values and those from the World Ocean Atlas climatology^45^.

For each profile, surface Chl-a concentrations (Chl[_Argo-Surf_]) were computed by averaging Chl-a data over the first optical depth. The first optical depth was computed as the euphotic depth (i.e. the depth at which PAR was 1% of its surface value^46^), divided by 4.6^47^. Integrated Chl-a values (Chl[_Argo-Int_]) were calculated by integrating Chl-a between the surface and the euphotic depth of each profile. This depth was chosen to represent phytoplankton biomass situated within the epipelagic, photic zone. The Mixed Layer Depth (MLD) was computed using the threshold method with a density gradient criterion of 0.03 kg m^−3^, compared to the density at 10 m^48^. To evaluate the level of stratification within the water column (see Fig. 3), we computed the Brunt–Väisälä (buoyancy) frequency using the “*sw_bfrq”* function from MATLAB’s SEAWATER toolkit^49^, which utilises measurements of pressure, temperature and salinity, from the BGC-Argo float, to calculate the mid-depth Brunt–Väisälä frequency.

### Matchup data between remotely-sensed and BGC-Argo datasets

Version 3.1 of the European Space Agency’s Ocean Colour Climate Change Initiative (ESA OC-CCI)^50,51^ was used in this study. The OC-CCI product consists of merged and bias-corrected Chl-a data from the Sea-Viewing Wide Field-of-View Sensor (SeaWiFS), Moderate Resolution Imaging Spectroradiometer (MODIS), Medium Resolution Imaging Spectrometer (MERIS) and Visible Infrared Imaging Radiometer Suite (VIIRS) satellite sensors. Level 3, daily, mapped Chl-a data were acquired at a spatial resolution of 4 km from http://www.esa-oceancolour-cci.org. *Brewin et al*.^52,53^ and *Racault et al*.^28^ have shown that both standard ocean-colour algorithms and the OC-CCI algorithm perform relatively well in the Red Sea, supporting the use of satellite-derived Chl-a datasets. Previous studies in the Red Sea have also demonstrated that the OC-CCI product is characterised by significantly higher data availability in comparison to single-sensor-based missions^28^. Thus, we believe that the OC-CCI dataset is an optimum choice for phenological analysis. We refer the reader to the OC-CCI Product User Guide at http://www.esa-oceancolour-cci.org/?q=webfm_send/318 for a more extensive overview of data processing, sensor merging and uncertainty quantification.

Satellite Chl-a data (Chl[_Sat-Surf_]) were temporally matched to BGC-Argo data based on the BGC-Argo sampling date. For the phenology analysis, 5-day composites were calculated to reduce the number of missing data and increase the matchups available to compare phenological metrics estimated using Bio-Argo and satellite datasets. Spatial matchups were acquired by locating the closest 4 km pixel (nearest latitude and longitude) to the BGC-Argo sampling location, and computing the average of 3 pixels longitudinally, centred on that 4 km pixel (grey shaded squares in Fig. 1). A total of 84 satellite matchups were obtained over the sampling period (September 30^th^ 2015 to September 27^th^ 2016).

### Computation of phytoplankton phenology metrics

Following the approach published in *Racault et al*.^28^, we utilised the cumulative sums of anomalies method, based on a threshold criterion, to estimate phytoplankton phenology metrics from both satellite-derived and BGC-Argo Chl-a datasets. This method has previously been applied for investigating phenology from satellite-derived climatology and interannual time-series in the Red Sea^23,28^, and *in situ* glider-based seasonal time-series in the Southern Ocean^16^. Due to the varying sampling frequency of the float and coverage of the satellite, we calculated, for both the BGC-Argo and the OC-CCI datasets, the average Chl-a concentration within 5-day periods, or so-called 5-day composites, which allowed us to generate temporally consistent and complete seasonal cycles. Generating complete Chl-a time series is important because the computation of phenology metrics can be impacted by the presence of missing data in the Chl-a time series^54,55^. However, even though averaging over 5 days reduces the resolution at which events in the phytoplankton growth period can be estimated, it does not significantly affect the spatial pattern of the estimated phenological metrics^56^. The cumulative sum of anomalies method requires a complete (i.e. gap-free) Chl-a time-series as an input, otherwise the phenology metrics cannot be calculated. Hence, to further improve the coverage of Chl-a satellite data, we applied a linear interpolation method that fills gaps in the time series. The interpolation method used is based on the MATLAB subroutine *inpaint_nans*, which interpolates missing data using a linear least squares approach^57^.

Next, we defined the threshold criterion as the median of the time series plus 5%^7,58,59^. Using this threshold, Chl-a anomalies were computed by subtracting the threshold criterion from the time series. The cumulative sums of anomalies were then calculated. Increasing (decreasing) trends in the cumulative sums of anomalies represent periods when Chl-a concentrations are above (below) the threshold criterion. The gradient of the cumulative sums of anomalies was then used to identify the timing of the transition between increasing and decreasing trends^28^. The initiation of the main phytoplankton growth period corresponded to the 5-day period when Chl-a concentrations first rose above the threshold criterion (i.e. when the gradient of the time series first changed sign). The termination of the main phytoplankton growth period was computed as the time when the gradient first changed sign following the occurrence of the maximum Chl-a concentration in the time series (the growth peak). The total duration of the main phytoplankton growth period was calculated as the number of 5-day periods between the timings of initiation and termination.

### Potential biases

Although not accounted for in the analysis of this study, we note that the transportation of water masses via eddies and surface currents in the northern Red Sea may influence Chl-a concentrations via horizontal advective processes that act to redistribute material towards or away from the BGC-Argo profiling site^60^. However, such features are usually observed in the southern-central Red Sea, as opposed to the northernmost region, which tends to be more convection-dependant (*Raitsos et al*.^61^, their Figs 3 and 4)^62^. Overall, our results infer that the BGC-Argo float generally captured the seasonal convective mixing that characterises the region.

We also note that variations in Chl-a concentration are not always associated with changes in biomass, but may result from fluctuations in the concentration of intracellular pigments as a result of photoacclimation processes. As the concentration of Chl-a is not a perfect proxy of phytoplankton biomass, we acknowledge that photoacclimation to low light levels, particularly during MLD deepening events, could impact our analysis. We have investigated the potential impact of photoacclimation using measurements of particulate backscattering acquired by the BGC-Argo float. Particulate backscattering at 700 nm (b_bp_700) averaged over the first optical depth and the first euphotic depth, generally increases in conjunction with integrated and surface Chl-a concentrations (figure not shown). Thus, although photoacclimation likely exerts some influence on Chl-a concentrations under conditions of reduced light availability, we acknowledge that there is an overall increase in the concentration of particulate matter within the water column during the main phytoplankton growth period, indicating that there is a net increase in Chl-a associated with new production.

Finally, the choice of depth used for computing integrated Chl-a concentrations could potentially influence our analysis, particularly if we consider the fact that the SCM may occasionally reach depths that extend below the euphotic zone. In order to capture Chl-a variability in the deeper layers and verify that the winter increase in Chl-a concentrations observed at the surface is in fact new production associated with enhanced nutrient supply from convective mixing, we produced a time series of Chl-a concentrations integrated between 100 and 200 metres (Supplementary Fig. 2). During the main phytoplankton growth period, Chl-a integrated within the 100–200 metre depth range exhibits a significant increase between mid January and late February. Based on this, we can infer that the enhanced Chl-a detected by satellites at the surface is not just the redistribution of Chl-a from the SCM, but represents new production within the water column.

## Supplementary information

Supplementary Material

## Data Availability

The ESA OC-CCI satellite ocean-colour dataset used in this study is freely available at http://www.esa-oceancolour-cci.org. The PROVOR Biogeochemical-float dataset is freely available at http://www.argo.ucsd.edu and http://argo.jcommops.org.
