# Supplementary material for: Evaluating tropical phytoplankton phenology metrics using contemporary tools
Source: Sci Rep. 2019 Jan 24;9:674. doi: 10.1038/s41598-018-37370-4 (PMC6345824; doi:10.1038/s41598-018-37370-4)
Supplement: Supplementary file 1 — Supplementary Material [file 41598_2018_37370_MOESM1_ESM.pdf]

Evaluating tropical phytoplankton phenology metrics using contemporary tools

John A. Gittings [1], Dionysios. E. Raitsos [3, 5], Malika Kheireddine [2], Marie-Fanny Racault [3, 4], Hervé Claustre [6], Ibrahim Hoteit [1*]

[1] King Abdullah University of Science and Technology (KAUST), Department of Earth Science and Engineering, Thuwal, 23955-6900, Kingdom of Saudi Arabia

[2] King Abdullah University of Science and Technology (KAUST), Red Sea Research Centre, Biological and Environmental Science and Engineering Division, Thuwal, 23955-6900, Kingdom of Saudi Arabia

[3] Plymouth Marine Laboratory (PML), Remote Sensing Group, The Hoe, Plymouth, PL1 3DH, United Kingdom

[4] National Centre for Earth Observation (NCEO), Plymouth Marine Laboratory, Plymouth, PL1 3DH, United Kingdom

[5] Department of Biology, National and Kapodistrian University of Athens, Athens, Greece

[6] Laboratoire d'Océanographie de Villefranche, Marine Optics and Remote Sensing Laboratory, Villefranche-sur-Mer, France

*Correspondence should be directed to Ibrahim Hoteit (ibrahim.hoteit@kaust.edu.sa).

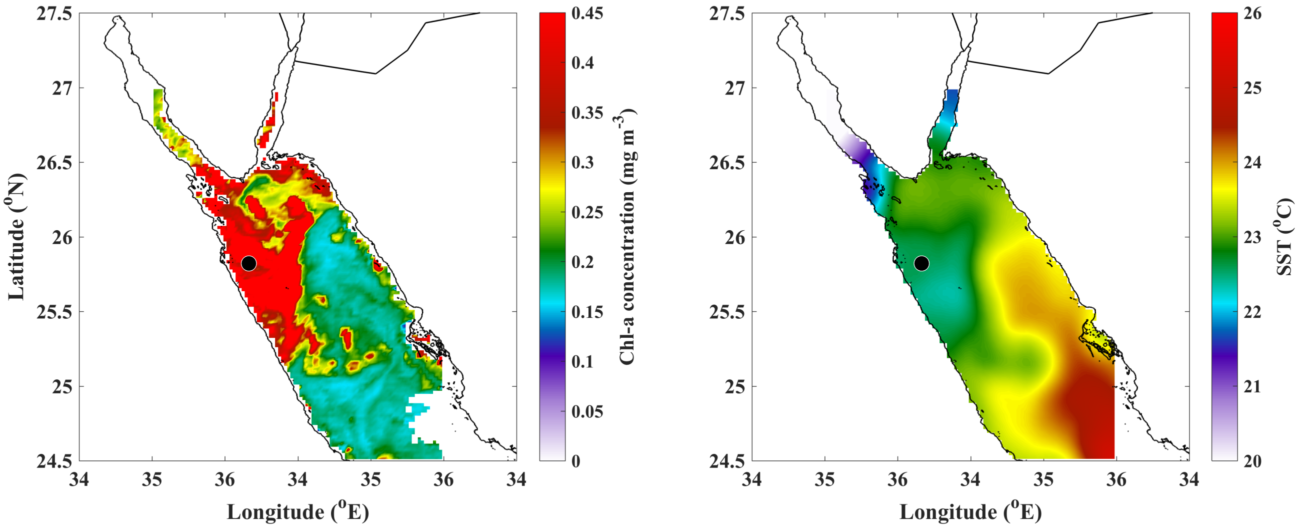

Supplementary Figure 1. Daily satellite imagery of Chl-a concentration (acquired from the ESA OC-CCI product, version 3.1) and Sea Surface Temperature (SST, NASA GHRSST OSTIA product), corresponding to the largest peak observed in Chl-a concentrations in late February. The precise date of the satellite imagery is 26th February 2016 and the black, filled circle highlights the position of the BGC-Argo float on this date. The float appears to be passing through a region of notably colder SST and high Chl-a concentrations, indicating the potential occurrence of convection-driven vertical mixing.

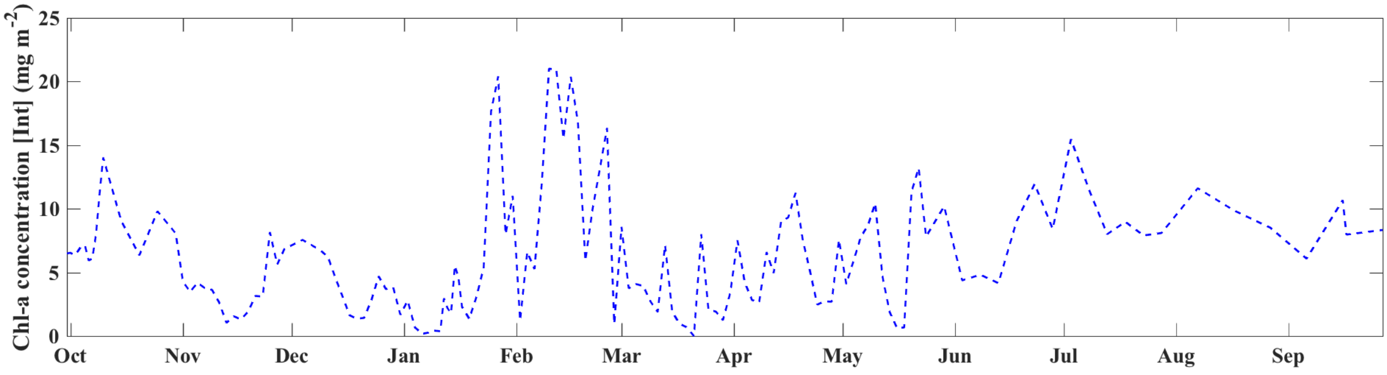

Supplementary Figure 2. Time series of BGC-Argo Chl-a concentrations integrated between 100 and 200 metres. Note that the time series represents the integrated Chl-a concentration for each BGC-Argo profile, and thus exhibits a temporal resolution that varies in accordance with the sampling frequency of the BGC-Argo float (1 to 10 days). Between January and February, during the main phytoplankton growth period, Chl-a concentrations integrated between 100 and 200 metres exhibit a marked increase, indicating an overall increase in new production within the water column.
